# Supplementary material for: Community-based surveillance of Chagas Disease: Characterization and use of Triatomine Information Posts (TIPs) in a high-risk area for triatomine reinfestation in Latin America
Source: PLoS Negl Trop Dis. 2025 Jun 23;19(6):e0013153. doi: 10.1371/journal.pntd.0013153 (PMC12208441; doi:10.1371/journal.pntd.0013153)
Supplement: S1 Table — (DOCX) [file pntd.0013153.s006.docx]

**S1 Table 1. Profile of participants in the different focus groups (FG) according to role and sex.**

| **Profile of Participants** | | | | | | |
| --- | --- | --- | --- | --- | --- | --- |
|  | **Professional role** | | | **Sex** | | **Total** |
|  | **Endemic disease coordinator** | **Others*** | **Not opinion** | **Men** | **Female** |  |
| **FG 1** | 8 | 2 | 0 | 7 | 3 | 10 |
| **FG 2** | 8 | 0 | 0 | 8 | 0 | 8 |
| **FG 3** | 5 | 1 | 0 | 1 | 5 | 6 |
| **FG 4** | 8 | 1 | 0 | 6 | 3 | 9 |
| **FG 5** | 11 | 0 | 0 | 9 | 2 | 11 |
| **Total:** | **40** | **4** | **0** | **31** | **13** | **44** |
| *Others: Supervisors and data entry clerks. | | | | | | |
