# Supplementary material for: Community-based surveillance of Chagas Disease: Characterization and use of Triatomine Information Posts (TIPs) in a high-risk area for triatomine reinfestation in Latin America
Source: PLoS Negl Trop Dis. 2025 Jun 23;19(6):e0013153. doi: 10.1371/journal.pntd.0013153 (PMC12208441; doi:10.1371/journal.pntd.0013153)
Supplement: S2 Table — FG: focus groups. (DOCX) [file pntd.0013153.s007.docx]

**S2 Table 2. Time in the position of endemics coordination.** FG: focus groups.

|  | **Time of Coordination** | | | | | | **Total** |
| --- | --- | --- | --- | --- | --- | --- | --- |
|  | **< 1 year** | **1-2 years** | **2-5 years** | **5-10 years** | **> 10 years** | **Blank** |  |
| **FG 1** | 3 | 1 | 1 | 0 | 3 | 0 | 8 |
| **FG 2** | 4 | 0 | 0 | 0 | 3 | 1 | 8 |
| **FG 3** | 1 | 0 | 1 | 2 | 1 | 0 | 5 |
| **FG 4** | 4 | 0 | 2 | 0 | 2 | 0 | 8 |
| **FG 5** | 7 | 0 | 2 | 0 | 2 | 0 | 11 |
| **Total:** | **19** | **1** | **6** | **2** | **11** | **1** | **40** |
