# Supplementary material for: Community-based surveillance of Chagas Disease: Characterization and use of Triatomine Information Posts (TIPs) in a high-risk area for triatomine reinfestation in Latin America
Source: PLoS Negl Trop Dis. 2025 Jun 23;19(6):e0013153. doi: 10.1371/journal.pntd.0013153 (PMC12208441; doi:10.1371/journal.pntd.0013153)
Supplement: S3 Table — (DOCX) [file pntd.0013153.s008.docx]

**S3 Table 3. Number of participants in the different focus groups (FG) who mentioned encountering triatomines in the territories.**

| **Groups** | **Yes** | **No** | **Does not know** | **No opinion** | **Total** |
| --- | --- | --- | --- | --- | --- |
| **FG 1** | 7 | 0 | 0 | 1 | 8 |
| **FG 2** | 8 | 0 | 0 | 0 | 8 |
| **FG 3** | 5 | 0 | 0 | 0 | 5 |
| **FG 4** | 8 | 0 | 0 | 0 | 8 |
| **FG 5** | 10 | 0 | 0 | 1 | 11 |
| **Total** | **38** | **0** | **0** | **2** | **40** |
