# Supplementary material for: Community-based surveillance of Chagas Disease: Characterization and use of Triatomine Information Posts (TIPs) in a high-risk area for triatomine reinfestation in Latin America
Source: PLoS Negl Trop Dis. 2025 Jun 23;19(6):e0013153. doi: 10.1371/journal.pntd.0013153 (PMC12208441; doi:10.1371/journal.pntd.0013153)
Supplement: Attachment — Submitted filename: Response to Reviewers.docx [file pntd.0013153.s012.docx]

Dear reviewers, we appreciate your suggestions and have accepted almost all of them. Below are the point-by-point responses.

Reviewer #1: The experimental design is correct and the results are based on clear statistical analysis. The discussion has been developed on the basis of the interpretation of the results.The authors have clearly mentioned the ethical endorsement made.

Reviewer #2: This study adopted a mixed-methods approach, with both objectives and approaches applied to health using an exploratory research design, encompassing both descriptive and analytical forms of research and is appropiate to adress the objectives, also de population and study area is appropiate described.

Reviewer #3: The methods would benefit from an overview of the research questions and study objectives and the steps taken to achieve each objective. These could be emphasized more throughout the whole paper and used to structure the paper.

**Authors**: We appreciate the comments and acknowledgments of the reviewers.

**Results:**
Reviewer #1: The results and analysis have been described correctly. There is an excessive use of figures and it is not clear which will be part of the text and which will be supplementary information.

**Authors**: We understand the reviewer's considerations. On the other hand, we present a comprehensive, complete, and thorough diagnosis of the PITs across a very large geographic area. In this sense, we believe that the number of figures is both adequate and necessary. Additionally, Reviewer 3 suggested incorporating tables from the supplementary material into the body of the text. To address your question about which figures are part of the main text or the supplementary material, we have highlighted the figures from the supplementary material in the marked-up version of the article.

Reviewer #2: The study mets all the parameters for this section

**Authors**: We appreciate your feedback

Reviewer #3: The results would also benefit from more structure. Please organize the results section by the research question/objective that each result answers, using the question or theme as a subheadings.

**Authors**: This organization has already been done. Due to the length of the article, it may have gone unnoticed by you. These topics can be found in the following lines:

**256- Physical, situational, structural, and spatial characterization of TIPs**

**423- TIPs located in common spaces of the FHS**

**479- Characterization of the use and productivity of TIPs**

**497-498 Perception of health surveillance professionals about the functioning of TIPs in the territories.**

Furthermore, adding subtopics makes reading more tiring, and the journal itself advises against it..

L504-509 Give a brief summary of each item listed

**Authors**: The information related to this supplementary material is detailed in the following paragraphs.

L526: Put information in table S3 into the main text
In general, much of the information that is stated to be in supplementary material would be better in the main text.

**Authors**: We consider your suggestion pertinent. However, the main text already contains a large number of figures, and one of the requests from Reviewer 1 was to remove some. Therefore, we chose not to add any more elements to the main text

In the perceptions of health surveillance professionals section, the quotes are valuable, but they are too numerous and too long, such that they lose their effectiveness. I suggest reducing the number of quotes and also reducing their length so that the point they are meant to illustrate is clearer. Also, more subheadings would be useful here to help guide the reader through the plethora of content.

**Authors**: We understand the reviewer's concern. In fact, the work is very dense, extensive, and presents a large amount of information. However, we made a selection and still left out several relevant statements and testimonies. The details included in the manuscript are essential for us to arrive at the summary presented in Frame 1. In this sense, we chose to keep all the reports in full.

**Conclusions:**

Reviewer #1: In the conclusions the authors detail the contributions of the study and the information and production undertaken to support the reactivation of TIPs, the access constraints created by the pandemic were well explained and the procedures used to address them.

**Authors**: We appreciate your feedback.

Reviewer #2: The study mets all the parameters for this section. In the PDF I suggest the inclusion of some similar studies in Colombia and Guatemala

**Authors**: We appreciate your feedback and have added the suggested references.

Reviewer #3: The conclusions are supported by the data. The discussion section lacks structure and would benefit from more thematic subheadings to guide the reader.

**Authors**: The discussion section has already been structured around the central topics of the article, namely:

**Line 869 Physical, situational, and spatial characterization of TIPs**

**Line 965 Characterization of the productivity and use of TIPs**

**Line 998 e 999 Perception of health surveillance professionals about the functioning of TIPs in the territories**

**Summary and General Comments:**

Reviewer #1: The study provides a reliable description of the operation, structure and use of TIPs in a highly endemic area of Chagas disease in Brazil since the 1990s. It is an important study because it evaluates, over a significant period of time, the entomological surveillance system that has been established as an effective tool in other countries. The successes and limitations that emerge over time will be of great value to control programmes in the region. The study falls within the scope of the journal, but for publication I suggest some adjustments to the authors.
Introduction: Line 86 mentions passive surveillance but does not describe it properly, I suggest the authors include a brief description of it.

**Authors**: We appreciate your comment. However, we would like to highlight that passive surveillance is surveillance with public participation (the two terms are synonymous) and is properly described between lines 91 and 96.

Methodology: Line 179 should be consistent in the use of numbers or letters.

**Authors**: We appreciate the observation and the adaptation of the information provided

Results:
The number of figures is excessive and it is not clear in the text which figures should be added and which should be included in the text. Authors are advised to keep the more complex figures in the text and the rest as supplementary information.
For example: Figures 6, 7 and 8 could be summarised in a table as well as Figures 10, 11 and 12.

**Authors**: We appreciate your suggestion and understand your concern. However, these figures are crucial in the presentation of the results, and we do not agree with converting them into tables. We believe that making this change would make the data more difficult for readers to visualize, thus worsening rather than improving the clarity of the manuscript.

General comments

Reviewer #1: The experimental design is correct and the results are based on clear statistical analysis. The discussion has been developed on the basis of the interpretation of the results.

**Authors**: We appreciate your feedback.

Reviewer #2: This is a good research with well presented methodology and results. I include some comments in the PDF and recommend minor revision

**Authors**: We appreciate the suggestions; all of them were taken into account.

Reviewer #3: This is an important evidence-based demonstration of the realities and challenges of Chagas disease surveillance. The information presented will be useful for future researchers. The manuscript text is well-written, but at times hard to follow because there is so much information presented and not enough structure to help the reader keep track of it. The impact of the paper will greatly increase if the authors insert guideposts into the paper in the form of clear objectives and/or research questions and then framing the methods, results, and discussion in terms of those objectives or themes. The paper would greatly benefit from more subheadings as well.

**Authors**: We thank you for your work and considerations. However, as mentioned above, we believe that we have used the ideal number of subsessions.

A second critical change that must be made is reduction of acronyms. The paper uses too many acronyms, making it impossible to follow in many paragraphs (VC, CD, PHU, CHA, RHO, RHS, EDCA, SISPCDCh, FHS, TIP, etc.) The number of acronyms used must be reduced to two or three at most.

**Authors**: Concordamos com o revisor e removemos a maioria das siglas — VC, CD, PHU, CHA , HU and FHS. No entanto, mantivemos as siglas que são importantes como identificadores do objeto, como PITs, SISPCDCh, etc. Isso ocorre porque o objeto é frequentemente reconhecido mais pela sigla do que pela palavra da qual se origina.
