# Supplementary material for: Community-based surveillance of Chagas Disease: Characterization and use of Triatomine Information Posts (TIPs) in a high-risk area for triatomine reinfestation in Latin America
Source: PLoS Negl Trop Dis. 2025 Jun 23;19(6):e0013153. doi: 10.1371/journal.pntd.0013153 (PMC12208441; doi:10.1371/journal.pntd.0013153)
Supplement: Attachment — Submitted filename: PNTD-D-24-01922_R1 Rev 20250504.docx [file pntd.0013153.s011.docx]

The presented manuscript describes information about the TIPS strategy for *T. cruzi* vector monitoring at the municipal level at Jequitinhonha Valley in the state of Minas Gerais, Brazil. This is a very interesting approach to evaluating how well the PNCDC is implemented among regions and proposing interventions based on scientific evidence. One of the best aspects of your manuscript is the discussion about PITs coverage, usage, and the disuse rate by establishment type.

The manuscript has merit for publication in PLOS NTD, however, a few revisions and corrections are required before acceptance.

**General Comments**

This manuscript brings to the scientific level very useful information to support public policies and propose interventions based on scientific evidence. Perhaps some aspects need to be better described, and some English proof reviews need to be done.

The manuscript is quite a bit longer than normally observed, and this is mainly due to the speech transcription included in the document. Please consider moving all the transcriptions to supplementary material. This way, the general readers interested will not be surprised by the number of your manuscript pages, and all the information will be easily available to the specific readers interested in the details on the transcriptions.

The presented manuscript is longer than normally observed. Please review and reduce most of the sections, especially the Results section.

Please review some of the acronyms used (eg. VC, PHU, RHS, etc...).

To consider making an English proof review of the document. For example, in the sentence:

“135 From this perspective, the community should direct any insects

136 suspected of being triatomines to the designated surveillance locations in their

137 countries: in Brazil, to the TIPs, as mentioned above.”

Consider: “From this perspective, the community should address any insects to the designated surveillance locations within their respective countries; in Brazil, for instance, to the TIPs, as previously mentioned.”

**Abstract**

I suggest you make the main objective clear in the abstract. When you use the sentence:

30 “For the first time in

31 scientific literature, the operational characterization, usage, and structuring of TIPs are

32 analyzed, alongside identifying factors hindering their sustainability.”

You are calling attention to the primacy of your study, and this is quite fine; perhaps, you do not inform us the main objective of your study clear at this section.

**Author Summary**

I suggest making an English proofread of this section. As an example, see the sentence above:

“55 In Brazil, when the community finds insects suspected of being triatomines, vectors

56 of the agent that causes Chagas Diseae (CD), they should take them to a Triatomine

57 Information Center (TIP), which can be in schools, basic health units, etc.”

Consider this revision:

“In Brazil, when the community finds insects suspected of being triatomines, the insects vectors of the agent that causes Chagas Disease (CD), they should take them to a Triatomine Information Center (TIP), which may be located in public infrastructure, like schools, basic health units, or other community facilities.”

**Introduction**

You have a very well-designed section, describing important information on the T. cruzi vector surveillance and the TIPs strategy of monitoring. Perhaps, try to be so concise in other sections of the manuscript.

Line 75: Chagas, 1909² to (Chagas, 1909)².

Materials and Methods

Specific Comments

Lines 66-71: "Their circadian activity and tolerance to human presence was used as a measure of establishment..." – Consider moving this information to the Methods section, as it seems out of place at the end of the Introduction.

**Results**

This section is fine and informative, perhaps once your manuscript is quite long, please consider reviewing and reduce this section, and then I can provide a detailed revision of the sentences.

Please consider moving the sentence above to the Introduction section.

“268 The implementation of TIPs in the health regions began in 1999 in the

269 Januária RHO, accounting for 26.4% of the total TIP installations in the study area.

270 Between 2000 and 2010, the process intensified in the Pedra Azul, Pirapora, and

271 Unaí RHOs regions, where 39.4% of the installations occurred. In the more recent

272 period, from 2011 to 2020, the Montes Claros RHS recorded 34.2% of TIP

273 installations in its municipalities.”

Please consider moving all the transcriptions to supplementary material.

**Discussion**

This section is fine and informative, perhaps once your manuscript is quite long, please consider reviewing and reduce this section, and then I can provide a detailed revision of the sentences.

**Tables and Frames**

All the tables need to be formatted as PNTD suggests (see <https://journals.plos.org/plosntds/article/figure?id=10.1371/journal.pntd.0008359.t001>).

**Figures**

You have an excessive number of figures. Please, consider grouping most of them into thematic figures.
