# Supplementary material for: Community-based surveillance of Chagas Disease: Characterization and use of Triatomine Information Posts (TIPs) in a high-risk area for triatomine reinfestation in Latin America
Source: PLoS Negl Trop Dis. 2025 Jun 23;19(6):e0013153. doi: 10.1371/journal.pntd.0013153 (PMC12208441; doi:10.1371/journal.pntd.0013153)
Supplement: Attachment — Submitted filename: Response_to_Reviewers_auresp_2.docx [file pntd.0013153.s013.docx]

Dear reviewers,

We appreciate your suggestions and accept some of them. Below are our point-by-point responses.

Reviewer #1: The experimental design is correct and the results are based on clear statistical analysis. The discussion has been developed on the basis of the interpretation of the results.The authors have clearly mentioned the ethical endorsement made.

Reviewer #4: This work is a case study with both qualitative and quantitative approaches that analyzes the physical, structural, situational, and spatial characteristics of the TIPs (Territories of Health Promotion), the productivity and engagement of the population, the relevance of the Family Health Strategy in health surveillance with popular participation, and the perceptions of health professionals regarding the functioning and sustainability of the TIPs.

The study presents a research design that is appropriate for addressing the proposed objectives. The study population is clearly described, and the sample size appears sufficient to support the intended analysis.

However, the statistical methods proposed for analyzing the data are quite basic. The study does not include any advanced analytical strategies that could model the relationships among key variables such as the conditions of the TIPs, productivity levels, environmental contexts, or professional perceptions. In this sense, the study is limited to simple comparisons to meet its objectives, which restricts the depth of its analytical insights.

This study was approved by the Research Ethics Committee of the René Rachou Institute.

Reviewer #5: Yes, the objectives of the study is clearly articulated with a clear testable hypothesis;
Yes, the study is design appropriate to address the stated objectives;
Yes, the population is clearly described and appropriate for the tested hypothesis;
Yes, the sample size is sufficient to ensure adequate power to address the tested hypothesis;
Yes, The correct statistical analysis was used to support the conclusions;
Yes, all the concerns about ethical or regulatory requirements are being met.

**Authors**: We appreciate the reviewers' comments and recognition of the scientific merit of the manuscript.

Dear Reviewer #4: We appreciate your comments regarding the statistical analyses. We would like to highlight that, for the objectives of the study, the statistical analyses, even though simple, were able to achieve the objectives the research goals. We understand your consideration about the use of statistical models, which could broaden the perspective and depth of the findings. However, although However, although we utilized models (GEE, GLM etc.) in other studies, we did not have prior hypotheses that would justify the use and necessity of any particular model in the present study.

It is also important to emphasize that, even though simple, the chi-square test of independence was able to support our hypothesis that PITs installed in Basic Health Units remain more active than those installed elsewhere. In this sense, we disagree with the reviewer regarding the superficiality of our analyses.

**Results**

Reviewer #1: The results and analysis have been described correctly. Analysis has been developed on the basis of the interpretation of the results.

Reviewer #4: The analysis presented aligns with the general objectives of the study, particularly in describing and comparing key characteristics of the TIPs, community engagement, and professional perceptions. However, while the study adopts both qualitative and quantitative approaches, the quantitative analysis is limited to basic descriptive and comparative methods. No advanced statistical modeling or in-depth analytical strategies are included, despite the potential for richer insights through such methods. Therefore, although the analysis matches the general plan, it does not fully exploit the possibilities offered by the data collected.
The results are presented clearly in terms of basic findings and descriptive patterns. The study adequately describe and reports the physical and structural situation of the TIPs, the location of the TIPs and the characterization of the use and productivity the TIPs. However, the completeness of the results is limited by the simplicity of the analytical methods. Without deeper modeling or exploration of relationships between variables, some potentially important insights—such as factors influencing TIP sustainability or the impact of community engagement—are left underexplored. While the results are clearly presented, the analysis does not fully leverage the available data. The study relies on basic descriptive comparisons, which limits its ability to explore deeper relationships and extract more nuanced insights from the dataset.
The tables and images presented in the study are of adequate quality, being clear, well-organized, and properly labeled.

Reviewer #5: Yes, the analysis presented match the analysis plan;
Yes, the results are clearly;
No, the manuscpript has more figures and tables than necessary

**Authors**: We appreciate the comments of the reviewers.

Dear Reviewer #4: once again we thank you for your comments regarding the potential application of statistical models to the data obtained in the study. However, as already mentioned in the Methods section, we, the authors, believe that all our objectives and questions were sufficiently addressed through the methods and analyses employed, even if simple (in fact, this aspect was also highlighted by you). We did not have any questions to answer that required the use of models. We followed the classic research approach: question/objective versus method/analysis, without incorporating or expanding issues retrospectively.

Dear Reviewer #5: We understand the reviewer's considerations. On the other hand, we present a comprehensive, complete, and thorough diagnosis of the PITs across a very large geographic area. In this sense, we believe that the number of figures is both adequate and necessary.

**Conclusions**
 Reviewer #1: In the conclusions the authors detail the contributions of the study and the information and production undertaken to support the reactivation of TIPs, the access constraints created by the pandemic were well explained and the procedures used to address them.

Reviewer #4: The conclusions of the work align with the data presented but are limited to the analyses conducted. The authors touch on the key aspects of the data and its relevance, but they do not delve deeply into how the findings could contribute to a broader understanding of the topic. The study primarily focuses on descriptive analysis rather than using the data to expand our understanding of the subject at a deeper level. The study addresses public health relevance, particularly through the analysis of the Family Health Strategy and community engagement in health surveillance. The data highlight the importance of these elements in strengthening health interventions and improving the sustainability of health promotion initiatives.

Reviewer #5: Yes, the conclusions is supported by the data presented;
Yes, The limitations are described;
Yes, the authors do discuss how these data can be helpful to advance our understanding of the topic under study;
Yes, the public health relevance is addressed?

**Authors:** We appreciate the comments and recognition from Reviewers 1 and 5. Regarding Reviewer 4, we regret disappointing you once again. However, since we did not perform modeling analyses, as highlighted by your in other sections, we cannot draw any conclusions beyond those presented in the study. Nonetheless, it is important and necessary to emphasize that we discussed and concluded everything we proposed to investigate in the study, as noted by all the other four reviewers.

**General Comments**

Reviewer #4: Regional Health Offices (RHOs) and Regional Health Superintendence (RHS): What are the differences between them and which regions do they cover?

**Authors:** Dear reviewer, thank you for your question. The main difference between a RHOs and a RHS lies in their roles and levels of responsibility within the Unified Health System of Brazil. While RHS are management units that plan, implement, and monitor health policies and actions in their respective regions; RHS support in executing health policies and actions, coordinating and overseeing the activities of health services in the region. In summary, while RHS operate in a more strategic manner, setting guidelines and objectives for health in the region with a focus on coordination and integration of actions; RHOs work in a more operational capacity, monitoring the implementation of health policies, ensuring the quality and efficiency of services. Regarding coverage, the RHOs typically have a smaller number of municipalities, while the RHSs encompass a larger number of municipalities.

This sounds more like a figure legend than results:
The 337 number of TIPs installed in each municipality follows a similar color pattern described in Fig 3. Municipalities with one to five TIPs installed are highlighted in yellow, six to 10 in light orange, 11 to 15 in dark orange, and 16 to 19 in brown. Municipalities with no TIPs installed in that zone are shown in white.

Excessive figures, should be combined among them.

Participan, coordinators comments could be moved to supplementary material and not included in the main results of the study.

**Authors:** Dear reviewer, we appreciate your comments and even agree with some points you raised. However, we clarify that we did not find a better way to present the results shown on the map nor an appropriate combination for the figures. Regarding the removal of the statements from the main text, we are completely opposed to this suggestion, as they are the primary data related to the perceptions of health professionals. By analogy with quantitative data, they would be equivalent to graphs and tables. We are very sorry, but we were not able to address any of your suggestions.

Reviewer #5:  This manuscript brings to the scientific level very useful information to support public policies and propose interventions based on scientific evidence. Perhaps some aspects need to be better described, and some English proof reviews need to be done.

**Authors:** We sincerely appreciate your valuable comment and recognition of the scientific merit of the article. Regarding the English language, we apologize if the writing style and language used did not meet your expectations. We would like to clarify that a Brazilian company specialized in scientific English editing always reviews our manuscripts, and this is the first time we have received a critique regarding the language. We apologize once again, but unfortunately, we will not be able to implement the language improvements you suggested, except for the phrase change suggestions you provided, which we accept all.

Reviewer #5: The manuscript is quite a bit longer than normally observed, and this is mainly due to the speech transcription included in the document. Please consider moving all the transcriptions to supplementary material. This way, the general readers interested will not be surprised by the number of your manuscript pages, and all the information will be easily available to the specific readers interested in the details on the transcriptions.
The presented manuscript is longer than normally observed. Please review and reduce most of the sections, especially the Results section.

**Authors:** Dear reviewer, I appreciate your comments. As we have already mentioned to reviewer 4, we do not agree with transferring the transcript statements to supplementary material, as they are primary data and of fundamental importance to the manuscript. In this sense, really, it is indeed a manuscript longer than the conventional one.

Please review some of the acronyms used (eg. VC, PHU, RHS, etc...).

**Authors:** Dear reviewer, we appreciate your concern regarding the abbreviations. Reviewer 3, in a previous round, had requested that we remove the abbreviations, and we promptly complied. Therefore, the manuscript now contains abbreviations that are even more commonly used than the words they represent. We apologize, but we did not see it as essential to remove them.

To consider making an English proof review of the document. For example, in the sentence:
“135 From this perspective, the community should direct any insects
136 suspected of being triatomines to the designated surveillance locations in their
137 countries: in Brazil, to the TIPs, as mentioned above.”
Consider: “From this perspective, the community should address any insects to the designated surveillance locations within their respective countries; in Brazil, for instance, to the TIPs, as previously mentioned.”

**Authors:** we thank you for your excellent contribution and we accept it.

Abstract
I suggest you make the main objective clear in the abstract. When you use the sentence:
30 “For the first time in
31 scientific literature, the operational characterization, usage, and structuring of TIPs are
32 analyzed, alongside identifying factors hindering their sustainability.”
You are calling attention to the primacy of your study, and this is quite fine; perhaps, you do not inform us the main objective of your study clear at this section.

**Authors:** We agree with your observation and have revised the text.

Author Summary

I suggest making an English proofread of this section. As an example, see the sentence above:

“55 In Brazil, when the community finds insects suspected of being triatomines, vectors
56 of the agent that causes Chagas Diseae (CD), they should take them to a Triatomine
57 Information Center (TIP), which can be in schools, basic health units, etc.”
Consider this revision:

“In Brazil, when the community finds insects suspected of being triatomines, the insects vectors of the agent that causes Chagas Disease (CD), they should take them to a Triatomine Information Center (TIP), which may be located in public infrastructure, like schools, basic health units, or other community facilities.”

**Authors:** Thank you once again, and we accept your suggestion.

Introduction
You have a very well-designed section, describing important information on the T. cruzi vector surveillance and the TIPs strategy of monitoring. Perhaps, try to be so concise in other sections of the manuscript.
Line 75: Chagas, 1909² to (Chagas, 1909)².

**Authors:** We appreciate your observation. However, according to zoological nomenclature rules, the author's name of the species described by him comes immediately after the scientific name, followed by a comma and the date of description. Here is the reference https://www.iczn.org/the-code/the-international-code-of-zoological-nomenclature/

Materials and Methods

Lines 66-71: "Their circadian activity and tolerance to human presence was used as a measure of establishment..." – Consider moving this information to the Methods section, as it seems out of place at the end of the Introduction.

**Authors:** Dear reviewer, we believe there was some misunderstanding, as this sentence is not present in the manuscript.

Results
This section is fine and informative, perhaps once your manuscript is quite long, please consider reviewing and reduce this section, and then I can provide a detailed revision of the sentences.
Please consider moving the sentence above to the Introduction section.
“268 The implementation of TIPs in the health regions began in 1999 in the
269 Januária RHO, accounting for 26.4% of the total TIP installations in the study area.
270 Between 2000 and 2010, the process intensified in the Pedra Azul, Pirapora, and
271 Unaí RHOs regions, where 39.4% of the installations occurred. In the more recent
272 period, from 2011 to 2020, the Montes Claros RHS recorded 34.2% of TIP
273 installations in its municipalities.”
Please consider moving all the transcriptions to supplementary material.

**Authors:** We appreciate your suggestions. However, the information you mentioned was obtained through this study; we collected it via a questionnaire sent during the data collection process. Therefore, we believe it is part of our results and it would not make sense to move it to the introduction. Regarding the transcriptions of the speeches, as previously mentioned, they are a primary result and of great importance to the study. Thus, we consider that they should remain in the main body of the article, even if this makes it quite lengthy.

Discussion
This section is fine and informative, perhaps once your manuscript is quite long, please consider reviewing and reduce this section, and then I can provide a detailed revision of the sentences.
**Authors:** We appreciate your comments and valuable contributions. Indeed, the discussion section, like the entire article, is quite lengthy. However, we need to highlight that we worked on a very extensive area (123 municipalities from different health regions) and used various methodological approaches. In this sense, we delivered a comprehensive and extensive work, which makes it unfeasible to reduce the sections of the manuscript. We believe that such a reduction would significantly impact the quality of the manuscript and undermine the considerable effort involved in conducting and completing the entire study

Tables and Frames
All the tables need to be formatted as PNTD suggests (see https://journals.plos.org/plosntds/article/figure?id=10.1371/journal.pntd.0008359.t001).
**Authors:** We appreciate your comments. The tables have been duly reviewed.

Figures
You have an excessive number of figures. Please, consider grouping most of them into thematic figures.

**Authors:** Dear reviewer, we appreciate your comments. However, we clarify that we did not find a better way to appropriate combination for the figures.

Use this section to provide overall comments, discuss strengths/weaknesses of the study, novelty, significance, general execution and scholarship. You may also include additional comments for the author, including concerns about dual publication, research ethics, or publication ethics. If requesting major revision, please articulate the new experiments that are needed.

Reviewer #1: The study provides a reliable description of the operation, structure and use of TIPs in a highly endemic area of Chagas disease in Brazil since the 1990s. It is an important study because it evaluates, over a significant period of time, the entomological surveillance system that has been established as an effective tool in other countries. The successes and limitations that emerge over time will be of great value to
control programmes in the region. The experimental design is correct and the results are based on clear statistical analysis. The discussion has been developed on the basis of the interpretation of the results. The study falls within the scope of the journal, and adjustments were appropriately realized.I had suggested reducing the figures, but after the explanation given by the authors, I consider that the tables and figures should be included as proposed by them.

**Authors**: Once again, we thank the reviewer for their work and excellent contributions, as well as for recognizing the scientific merit of the study.

Reviewer #4: The study provides an analysis of TIPs using a mixed-methods approach, focusing on their physical, structural, situational, and spatial characteristics, as well as community engagement, the Family Health Strategy, and health professionals' perceptions regarding the sustainability of TIPs.

The study’s objectives are clearly defined, and appropriate methods are applied for data collection and analysis. Additionally, it addresses a relevant public health issue, particularly concerning community participation and the Family Health Strategy in improving health surveillance. The tables and figures are well-organized and contribute to the clarity of the presented results.

However, the data analysis is limited, as it primarily relies on descriptive statistics, which restricts the ability to explore the relationships between the studied factors in depth. The use of more advanced analytical techniques would provide a more nuanced view of the data. Moreover, the manuscript is excessively long, with a large number of figures that could be reduced or combined to avoid redundancy. The results and discussion sections could also be more concise, focusing on the most relevant findings and their impact.

Although the study highlights the relevance of TIPs for public health, it does not fully explore how its findings could contribute to advancing theoretical and practical knowledge in the field. A more extensive discussion of the implications of the findings for public policy and future research directions would strengthen the study's impact.

Overall, the study is well-executed in terms of data collection, but the lack of depth in analysis and the need to reduce the length and redundancy of the text limit its clarity and focus. Incorporating more complex analytical techniques and condensing the redundant sections would improve its academic rigor and practical relevance. Additionally, it would be helpful to include policy recommendations based on the findings and suggest directions for future research on TIP sustainability in different contexts.

**Authors**: Dear reviewer, we appreciate the thorough and careful reading of our manuscript. We find the perspective you present regarding the potential contained in the data for creation and analysis in models to be very interesting. We would like to highlight that these considerations will be incorporated into future studies; however, for this current work, they will not be considered. The reasons are: we believe that all objectives have been adequately addressed through the analyses employed. The use of models would introduce new questions that were not our primary goal in this study. Regarding the transcriptions of the speeches, we consider them to be among the most important results of the work. Therefore, we do not agree with moving them to supplementary material. Finally, concerning the discussion of public health policies and perspectives arising from the results obtained, we agree that there is great potential in our work for this discussion. However, we limited ourselves to discussing the results in detail, from a scientific perspective, which made the discussion very extensive, preventing the addition of other perspectives. We thank you once again and ask for your understanding regarding the scope and approach we adopted in the manuscript.

Reviewer #5:  The presented manuscript describes information about the TIPS strategy for T. cruzi vector monitoring at the municipal level at Jequitinhonha Valley in the state of Minas Gerais, Brazil. This is a very interesting approach to evaluating how well the PNCDC is implemented among regions and proposing interventions based on scientific evidence. One of the best aspects of your manuscript is the discussion about PITs coverage, usage, and the disuse rate by establishment type.

**Authors**: Dear reviewer, we thank you once again for your thorough and valuable contributions. We accept many of your suggestions and are confident that the manuscript's quality has been enhanced through them.
